# The use of music as an arts-based method in migrant health research: a scoping review protocol

**DOI:** 10.12688/hrbopenres.13121.1

**Published:** 2020-10-12

**Authors:** Fran Garry, Sylvia Murphy Tighe, Anne MacFarlane, Helen Phelan

**Affiliations:** 1 Irish World Academy of Music and Dance, University of Limerick, Limerick, V94 T9PX, Ireland; 2 Department of Nursing & Midwifery and Health Research Institute (HRI), University of Limerick, Limerick, V94 T9PX, Ireland; 3 Public & Patient Involvement Research Unit, Graduate Entry Medical School and Health Research Institute (HRI), University of Limerick, Limerick, V94 T9PX, Ireland; 4 Irish World Academy of Music and Dance and Health Research Institute (HRI), University of Limerick, Limerick, V94 T9PX, Ireland

**Keywords:** Music, migrant, health, well-being, arts-based research

## Abstract

There is increasing recognition that people’s lived experience needs to be incorporated into health decision-making. This has led to rising imperatives for involving the public in health processes, including research. While there have been significant advances in the field, patterns of exclusion still exist in some areas, including migrant participation in health research. Migration and mobility create challenges around social inclusion and this extends to social and cultural practices used in research. There is an emerging body of literature about improving meaningful, participatory spaces for migrants’ involvement in health research using creative tools and techniques that are attuned to cultural diversity. These include the use of arts-based research methods. There is strong evidence for the use of music, particularly singing, as an effective arts-based participatory **tool *.* The goal of this scoping review is to investigate the evidence for the use of music as an arts-based method in migrant health research. Developed by an interdisciplinary team specialising in public and patient involvement; nursing and midwifery; primary health care; and the performing arts, it aims to analyse existing evidence across disciplines that are not usually studied together, identify gaps in current knowledge and use these as a foundation to build effective strategies towards increasing access to and knowledge of participatory, arts-based methods using music in migrant health research.

**Methods:** The protocol for this scoping review follows the guidelines and stages set out in the JBI Reviewer’s Manual ( *Peters et al., *2017), and by Levac *et al*, (2010), which build on the methodological framework of Arksey and O’Malley (2005). This incorporates six stages: 1) Identifying the research question; 2) Identifying relevant studies; 3) Study selection; 4) Charting the data; 5) Collating, summarising, and reporting results; and 6) Consultation.

## Introduction

There are rising imperatives for involving the public in health decision-making. The rationale comes from several sources ( Gibson *et al.*, 2012) but includes an increasing recognition that people have lived experience of health and, thus, are experts whose experiential knowledge needs to be incorporated into analysis to ensure a comprehensive epistemic baseline ( Popay & Williams, 2006). This patient and public knowledge has a key role to play in the promotion of good health; ( Ascenso *et al.*, 2018; Brown *et al.*, 2018; Jones *et al.*, 2013; Roberts *et al.*, 2017; Tarr *et al.*, 2014) prevention of ill health; ( Chabot *et al.*, 2019; Conceição *et al.*, 2016; Fancourt & Perkins, 2018a) managing and treating illness; ( Cao *et al.*, 2016; Gopalkrishnan 2016; Wan *et al.*, 2010) the development of health services and strategy ( WHO Europe, 2002; WHO, 2018) as well as health research ( HRB Strategy 2016-2020; Horizon 2020; Ramsden *et al.*, 2010; Wallerstein *et al.*, 2018), which is the focus of this article.

There have been significant advances in the field of involving the public in health research. In spite of challenges with conceptualisation ( Sarrami-Foroushami *et al.*, 2014), there is an acknowledged need for expanding the evidence base around meaningful, rather than tokenistic, involvement drawing on a variety of research traditions and methods ( Chew-Graham, 2015). There are specific areas that require further development including patterns of exclusion whereby migrant’s involvement in health decision-making is rare ( de Freitas & Martin, 2015; MacFarlane, 2019). However, there are complexities in accessing and understanding this experience. ‘Migrants’ are a heterogenous group of peoples. There is no agreed universal definition for a ‘migrant’. The broadest one, from the International Organisation for Migration is ‘anyone who has moved from their habitual residence (within or across borders)’. The term ‘migrant’ can refer to students, family reunification, labour migrants (moving to higher/lower paid employment), people seeking protection, people who are trafficked and so on. Further still, migrants’ identities and experiences are not only defined by this issue of ‘movement’ but, also, by gender, ethnicity, class and culture. Intersectionality captures this complexity ( Crenshaw, 1995). Finally, migrants have different health experiences but also share health experiences with the people in the communities in which they settled. While there are specific health implications of pre-migration and migration journeys (trauma, screening), migrants have non-communicable diseases and considerable health needs in relation to these, just like host populations ( WHO Europe, 2018).

There is a view that these patterns of exclusion exist because migrants are hard to reach or easy to ignore ( Lightbody, 2017). This view focuses on challenges in access and communication and concerns about the additional work and resources that health researchers would need to involve migrants. Certainly, there are issues to consider. These include cross-cultural interactions between migrants and health researchers ( O'Reilly-De Brún *et al.*, 2018); concerns for migrants about safely interacting with academia/officialdom ( van den Muijsenbergh *et al.*, 2016); and problems with representation of power, particularly given the heterogeneity of migrant experiences ( De Freitas *et al.*, 2014; Vaughn *et al.*, 2017).

Given the ethical imperatives for migrants’ involvement and the anticipated benefits for a more comprehensive knowledge base, which incorporates their expertise, a key question is how best to involve migrants in health research and, further, what is the evidence of best practice in terms of ethics, ontology and methodology? Migration and mobility create inevitable challenges around social inclusion and this extends to social and cultural practices used in research ( Rubina *et al.*, 2010). Therefore, in order to optimise migrants’ involvement in health research we need to think creatively and innovatively about effective strategies to enable and ensure meaningful participation. Participatory Health Research is a paradigm which centralises participation from the outset to the completion and dissemination of research. It emphasises shared governance and community ownership ( Wright, 2015). There is a rich tradition of participatory approaches that provide valuable tools, practices and techniques that have relevance for this endeavour ( MacFarlane, 2019).

We know that participatory spaces are shaped by using these tools, practices and techniques to create and support dialogues between stakeholders ( Cornwall & Schatten Coelho, 2007; Massey, 2005; Power, 2001). There is evidence that the use of inherently analytic and visual tools and techniques from the Participatory Learning and Action (PLA) research approach, for example, can be used to involve migrants in meaningful and impactful dialogues with stakeholders from universities and health care sectors ( de Brún *et al.*, 2017; O'Reilly-De Brún *et al.*, 2018). PLA does still, however, centre around knowledge sharing via language so it is valuable to think about other forms of communication and interaction that may be useful to complement or augment such approaches. Within music research, there is compelling evidence that certain musical practices, such as singing, are particularly effective in fostering participation through increasing empathy ( Ahlquist, 2006; Bithell, 2014; Clarke *et al.*, 2015; Magowan, 2019; Phelan, 2017a), accelerating interaction within new and diverse groups ( Pearce *et al.*, 2015) and facilitating multi-modal communication beyond the semantic limits of language ( Welch *et al.*, 2014). Studies demonstrate the value of music as a participatory strategy in a wide range of health and well-being areas including mother-infant bonding ( Cevasco, 2008; Fancourt & Perkins, 2017; Fancourt & Perkins, 2018b; Persico *et al.*, 2017), speech and language ( Brown *et al.*, 2018; Cogo-Moreira *et al.*, 2013; Flaugnacco *et al.*, 2015), pain management ( Chuang, 2018; Gokyildiz Surucu *et al.*, 2018), hypertension ( Cao *et al.*, 2016; do Amaral *et al.*, 2016; Kühlmann *et al.*, 2016), cognitive decline ( Gooding *et al.*, 2014; Strong & Mast, 2019), ageing and frailty ( Chabot *et al.*, 2019; Trombetti *et al.*, 2011; Ueda *et al.*, 2013), trauma and abuse ( Bronson *et al.*, 2018; Carr *et al.*, 2012; Landis-Shack *et al.*, 2017), surgery ( Dionigi & Gremigni, 2017; Jayakar & Alter, 2017; Nilsson, 2009), perinatal care ( Chang *et al.*, 2015b; Fancourt & Perkins, 2018b; McCaffrey *et al.*, 2020; Perkins *et al.*, 2018; Reilly *et al.*, 2019), physiotherapy ( Hsu *et al.*, 2016; Lim *et al.*, 2011), autism ( Cook *et al.*, 2018; LaGasse, 2017; Woodman *et al.*, 2018), cerebral palsy ( Alves-Pinto *et al.*, 2017; Bringas *et al.*, 2015; Marrades-Caballero, 2018), stroke ( Fogg-Rogers *et al.*, 2016; Forsblom *et al.*, 2010; Raglio *et al.*, 2017; Tamplin *et al.*, 2013), other acquired brain injuries ( Baker *et al.*, 2018; Baker *et al.*, 2019; Roddy *et al.*, 2018; Tamplin *et al.*, 2013), Parkinson’s Disease ( Barnish *et al.*, 2016; Di Benedetto *et al.*, 2009; Evans *et al.*, 2012; Han *et al.*, 2018; Harrison *et al.*, 2017; Stegemöller *et al.*, 2017; Tanner *et al.*, 2016), and dementia ( Chang *et al.*, 2015a; Daykin *et al.*, 2017; Dowlen *et al.*, 2018; Moss & O’Neill, 2019; Särkämö *et al.*, 2014). The specific literature around music and migrant health and well-being is also growing ( Balsnes, 2016; Cain *et al.*, 2020; Henderson *et al.*, 2017; Phelan, 2017b; Phelan *et al.*, 2017).

Despite the numerous publications on the role of music in health and well-being, it is striking that an examination of reviews to date on the use of arts-based methods in health identify very few studies using music as an arts-based *research method.* The inclusion of artistic practices as methodological tools in research is frequently labelled arts-based research (ABR). A useful definition of ABR is provided by Leavy as, “a set of methodological tools used by qualitative researchers across the disciplines during all phases of social research, including data collection, analysis, interpretation and representation … arts-based practices draw on literary writing, music, performance, dance, visual art, film and other mediums” ( Leavy, 2009, p.ix).

The systematic review of arts-based methods in health research by Fraser & al Sayah, (2011) identified 30 articles using a variety of arts-based methods including photography (11), drawing (10), theatre (7) and poetry (2) but none of the studies specifically mentioned music. Boydell *et al.* (2016)  identified 71 papers in their scoping review of arts-based health research using photography (23), theatre (21), drawing (8), film (8), poetry (3) and dance (1). Though eight studies used a variety of artistic practices, none of them mentioned music specifically. Coemans & Hannes, (2017) identified photography, theatre and poetry as the most often used artistic practices in ABR. Thus, while it is clear that music is being used as a tool of participation in health contexts, its role as a research tool is less well defined. A number of reasons may be suggested for this apparent anomaly.

Firstly, much of the research on the role of music in health and well-being is observational. For example, in the review carried out by Fraser and al Sayah, 30 articles were identified as using ABR but 56 were excluded as they did not use the artistic practice as part of the research method ( Fraser & al Sayah, 2011). Such research observes and reports on the impact of music in health and well-being contexts, but does not use the arts as a research tool. In the case of music, the music practice becomes an object of study, rather than an intrinsic part of the research design, implementation or interpretation. Participation may be an important element of the observed practice, but not necessarily an aspect of the research method.

Secondly, there is a lack of consensus concerning the terminology used to describe research through the arts. The term arts-based research was first coined in the early 1990’s by Elliot Eisner ( Eisner, 2008; Barone & Eisner, 2012) and while it has steadily grown in usage since that time, there is still a great deal of research through the arts (including research in areas such as music therapy, community music and music education) which does not use this terminology. This is especially the case in health research, which has adopted the use of ABR terminology more slowly than, for example, creative arts practices, education, community-based research ( Ledger & McCaffrey, 2015). The growing area of practice as research (PaR), also variously known as arts practice research, artistic research, arts practice research and practice-based research, further complicates the terrain ( Nelson, 2013; Smith & Dean, 2009). Debates regarding nuances of meaning have resulted in a plethora of terms which often confuse rather than clarify the discourse ( Chilton, 2013).

Another important factor includes understandings of what constitutes health research. The WHO definitions of health ( Fancourt & Finn, 2019, p.2) and art (ibid, p.1) note that health is inclusive of individual, social and cultural wellbeing and that artistic practices also extend across individual, social and cultural spaces. Blacking’s definition of music as ‘humanly organised sound’ (1973, p.10) was a direct response to narrower, ethnocentric definitions based on Western European aesthetics. These inclusive definitions challenge researchers to look beyond traditionally recognised health literature and disciplines to include health research carried out through music but published in areas such as ethnomusicology, cultural and social anthropology or community arts among others.

Finally, as Coemans and Hannes note, ABR is sometimes applied quite narrowly within the research design: “traditionally, ABR methods have been applied either as a data collection technique or as a dissemination technique” (2017, p.35). However, in ABR approaches, “the arts play a primary role in any or all of the steps of the research method” ( Austin & Forinash, 2005, p.458-459) in order to exploit “the capacities of expressive form to capture qualities of life that impact what we know and how we live” ( Barone & Eisner, 2012, p.5). A narrow application of the term often describes a process whereby artistic participation starts at the point of data generation or collection, but music is also used very successfully as a tool for initiating and creating partnerships for research. Research frameworks or reviews which do not utilise an explicitly articulated participatory approach often fail to capture or identify this music-based research as ABR.

For these reasons, a scoping review conducted by an interdisciplinary team which focuses specifically on *the use of music as an arts-based method* is both necessary and challenging. In order to develop optimal modes of participatory research using music as an arts-based method to inform the field of migrant health research, we need to analyse existing evidence across disciplines that are not usually studied together by health researchers, identify gaps in current knowledge and use these as a foundation to build effective strategies towards increasing access to and knowledge of participatory, arts-based methods using music in migrant health research. These approaches should apply our knowledge of music as a participatory strategy and adapt it for the purposes of research so that it is ethically guided to yield knowledge that cannot be accessed, analysed or represented through standard research frameworks and modes of communication ( Barone & Eisner, 2012). In addition, it is important to document the capacity of music to facilitate participatory spaces for building trust, raising participant voices in their own words and ways, and triggering empathy and understanding that leads to action ( Bartleet *et al.*, 2010; Bartleet & Higgins, 2018; Bonshor, 2018; Higgins *et al.*, 2010). This is very much in line with the aforementioned principles of PHR ( Wright, 2015).

### The purpose of the scoping review

The goal of this scoping review, therefore, is to investigate the evidence for the use of music as an arts-based method in migrant health research to advance knowledge about tools and techniques for participatory health research projects with migrants. In doing so, we have identified the following key objectives:

- Ascertain the extent of current publications using music in migrant health research and identifying itself as ABR.
- Identify the research stages (participating, generating data, analysing, interpreting, disseminating) in which music features as an ABR method or part thereof.
- Identify whether particular musical practices are used more than others as part of the research design and implementation.
- Identify the key strengths and challenges discussed in the literature around the use of music as a research tool in migrant health research.
- Identify gaps in current knowledge and use these as a foundation to build effective strategies towards increasing access to and knowledge of participatory, arts-based methods using music in migrant health research

Table 1 presents the definitions and boundaries that will guide how we utilise key concepts in our scoping review.

**Table 1.** Concepts, definitions and boundaries. Concepts, definitions and boundaries for undertaking a scoping review to investigate the use of music as an arts-based method in migrant health research.

Concepts of the research question	Definitions attached to the research question	Boundaries of the research question (inclusion & exclusion criteria)
**Arts-based** **research**	Arts-based research is “a set of methodological tools used by qualitative researchers across the disciplines during all phases of social research, including data collection, analysis, interpretation and representation … arts-based practices draw on literary writing, music, performance, dance, visual art, film and other mediums” ( Leavy, 2009, p.ix). Barone & Eisner (2012) define arts-based research as ‘an approach to research that exploits the capacities of expressive form to capture qualities of life that impact what we know and how we live’ (p.5).	Any research approach that integrates music in the research design, and self identifies as arts-based research is included. Observational research where music (as an intervention, activity or participatory practice) is the object of study but not part of the research method used to harness and extend knowledge of human experience is excluded.
**Music**	Blacking (1973) states "Music is a product of the behaviour of human groups, whether formal or informal: it is humanly organised sound. And, although different societies tend to have different ideas about what they regard as music, all definitions are based on some consensus of opinion about the principles on which the sounds of music should be organised."	Studies that focus on human music-making are included. Studies that focus on music-making by non- humans are excluded.
**Migrants**	A broad definition of a migrant, as used by the International Organisation for Migration, is “anyone who has moved from their habitual residence (within or across borders)”	Studies that include migrants are included. Studies that focus on community populations without any representation of migrants are excluded.
**Health**	WHO (1948) defines health holistically as “a state of complete physical, mental and social well-being and not merely the absence of disease or infirmity”, thus rooting health firmly within society and culture.	Studies that focus on physical, or mental or social well-being are included. Studies that are not focused on health are excluded.

## Methods

Due to the broad nature of the research question and objectives, we want to specifically capture the extent of the literature that utilises music as an ABR method in migrant health research in order to identify its evidence base. We aim to identify if and how music is being utilised as an ABR method in the field of migrant health research. With that in mind, a scoping review was identified as the most suitable methodology to help clarify key concepts and understand the extent of the evidence across disciplines around the use of music as an ABR tool in migrant health research. It is envisaged that a scoping review conducted in a systematic way can assist practitioners and policy makers in the area of migrant health research. To ensure rigour in our approach, the protocol for this scoping review follows the guidelines and stages set out in the JBI Reviewer’s Manual ( Peters *et al.*, 2017), and by Levac *et al.*, (2010), which consist of a further developed methodological framework from that of the widely cited Arksey & O’Malley (2005). The extended framework from Levac *et al.*, incorporates six stages: 1) Identifying the research question; 2) Identifying relevant studies; 3) Study selection; 4) Charting the data; 5) Collating, summarising, and reporting results; and 6) Consultation. This protocol will outline how we will address each of the six stages.

### Stage 1: Identifying the research question

In order to clearly identify the research question guiding the scope of the review, we iteratively searched and designed our key search terms to capture the most appropriate body of literature. In formulating our research question, we identified four key concepts that encompass the interdisciplinary nature of the scoping review. These are: ABR, music, health, and migrants. The principal contextual settings within which these concepts will be explored are: music as an ABR method, and migrant health. Our key objective is to use the literature to investigate if and how music of any genre (including singing, choirs, musical performance, solo music, ensemble music etc.) is being utilised as an ABR method in these contexts. The conceptualisation of novel or innovative approaches to health research and the utilisation of music as, or as a component of, an ABR method in research involving migrants is the focus of our enquiry. This led to the formulation of our research question: “What is the evidence for music as an arts-based method and its use in migrant health research?”

### Stage 2: Identifying relevant studies

Recognising that a systematic and comprehensive approach is a key strength of a scoping review, we want to ensure data sources are heterogenous with due attention across relevant disciplines, while not compromising feasibility. We will include grey literature such as theses/dissertations and reports. With that in mind, we are considering searches in a number of places including Scopus, Medline, Web of Science, CINAHL, Academic Search Complete, Social Science Premium Collection, PsychINFO, Springer LINK, Taylor & Francis Online, Google Advanced Search, and ProQuest Dissertations and Theses A& I. We can also refine the list as part of the iterative nature of our review process, and report on this process in the results paper. The bibliographical references of included papers and reviews on the topic will be checked for eligible articles that did not appear in the search. Deliberation among all members of the research team regarding exclusion and inclusion criteria at the outset of the scoping review process will occur. Table 2 provides an overview of the eligibility criteria for the scoping review.

**Table 2.** Eligibility criteria. An overview of the eligibility criteria for the scoping review.

Criterion	Inclusion	Exclusion	Justification
**Population** **and sample**	Human migrant	Any study population other than humans	Refers to human migrants of all ages
**Language**	Written in English	Any other language that is not in English	Reviewers only speak English
**Time period**	2009 to 2020	Any publications outside these dates	The publication of Leavy’s 2009 definition is the basis of our review
**Study focus**	Articles that discuss music as a participatory arts-based research method in migrant health	Articles that do not self- identify as arts-based research and utilise music as an arts-based method in migrant health	To build an evidence base for music as an arts-based method in migrant health research
**Type of** **article**	Peer reviewed journal articles, or peer reviewed books and chapters, or reviews and grey literature. Specifically, grey literature will include theses/dissertations, reports, conference proceedings, and editorials.	Any other literature that is not listed in the inclusion criteria, such as websites.	Scoping reviews aim to capture a comprehensive body of literature in order to expansively explore a broad research question. Preliminary searches of electronic databases and grey literature generally revealed papers reflecting our inclusion criteria Acknowledging feasibility and time constraints, we considered that the criteria listed would sufficiently capture the necessary literature to inform our review.
**Study** **location**	Any location – an international context.	None	Arts-based research has applications globally

### Stage 3: Search strategy and study selection

In line with Arksey & O’Malley’s (2005) framework, we will clearly define the terminology we intend to use within our search strategy in order to identify the most appropriate body of literature relevant to our research question. As noted by Levac *et al.* (2010), designing our search strategy has been an iterative process prioritising teamwork, interdisciplinary knowledge sharing, discussions and joint decisions around the definitions, concepts, and terminology involved. In order to test the appropriateness of selected databases and keywords, we are conducting preliminary searches in Taylor & Francis Online and CINAHL, searching article titles, abstracts, keywords and subject headings relating to our four key concepts: ABR, music, migrants and health. A comprehensive set of keywords will be used in the various databases encompassing the broad fields of arts, social science and health. If additional relevant keywords are identified, we will include these in our searches across all databases. Our search strategy will be further developed in collaboration with one of our faculty librarians.

Two independent reviewers will initially screen studies by title and abstract, and will subsequently conduct full-text reviews based on the pre-specified inclusion/exclusion criteria relevant to our research question. The two reviewers will meet at multiple stages of the review process to discuss any challenges, uncertainties or necessary refinements to the search strategy ( Levac *et al.*, 2010). If disagreements arise regarding inclusion or exclusion of studies, and cannot be resolved by consensus, a third reviewer will make the final decision. All eligible studies will be managed using Mendeley reference manager. Table 3 provides an overview of search terms.

**Table 3.** Search terms.

Study Population	Arts-based research (ABR)	Music	Health
asylum* “asylum seeker” refugee migrant migrat* emigrant emigrat* immigrant nomad foreigner displaced stateless state-less noncitizen non-citizen outsider newcomer “newly arrived” “new arrival” “recent entrant” non-national (title)	Arts-based Arts-informed “Arts practice” ABR	Music “Music listening” “Music practice” Singing Songs “Choral music” Choirs “Solo music” “Instrumental music” “Ensemble music” Songwriting “Musical performance”	Health Wellbeing “Physical health” “Mental health” “Physical wellbeing” “Mental wellbeing” “Social wellbeing” “Quality of life”

### Stage 4: Preliminary charting elements and associated questions

Following guidelines from the JBI Reviewer’s Manual, a tabular chart organised in Excel (see Table 4) will be used for data extraction. This is comprised of JBI recommended charting elements and associated questions to guide the charting process. Charting elements about interventions are not included as these are not the focus of the present review. The table also includes review specific rows that have been added to record how articles address core concepts of this review: ABR, music, migrants and health. These additional rows will support the next stage of the review process; collating, summarising, and reporting the results (identifying themes). The process of charting data will be iterative so that new data can be incorporated if it enhances charting and analysis for our review question. Table 4 provides a preliminary table of charting elements and associated questions for data.

**Table 4.** Preliminary table of charting elements and associated questions for data.

Charting elements	Associated questions
**Publication details**	
**Author(s)**	Who wrote the study/document?
**Year of publication**	What year was the study/document published?
**Origin/country of origin**	Where was the study/document conducted and/or published?
**Publication type**	What type of publication is this? (empirical study, book/book chapter or grey literature)
**General study details**	
**Aims/purpose**	What were the aims of the work reported in the study/document?
**Methodological design**	What methodological design was used for this study?
**Study population and sample size (if** **applicable)**	Who is the target population of the study (i.e. what migrant group(s) and how many (n) were included in the study?
**Methods**	What specific methods (e.g. qualitative, quantitative, combined methods) were used in this study?
**Key findings that relate specifically to the concept of ABR using music in migrant health research**	
**Is there a definition of ABR from the** **literature**	How does the study define ABR?
**What form(s) of music are** **employed?**	What are the forms of music that are used in the study? Is music employed with other art forms? Who delivered the music component?
**What stage of the research process** **is music employed?** • Design • Governance • Data generation • Data analysis • Data (re)presentation • Dissemination • Other	Is music used at one/several stages of the study? Are different forms of music used at different stages?
**Was there a detailed description of** **the migrant population?**	Were socio-demographic details provided? Was the sample comprised of migrants and host country participants or solely of migrants? Is a definition of the migrant population provided?
**What was the research setting?**	Was it a clinical setting? Was it a community-based setting? Was it an educational setting?
**Who were the study team?**	Was a music specialist part of the research team/involved in delivering the music aspect of the study. If so, what kind of specialist? Therapist? Community musician? Professional musician? Artist/researcher? Were there partners from academic/health/NGO sectors? Had they worked together in research/participation projects prior the study? Will they continue to work together after the study?
**Was there consideration of ethical** **issues?**	Is there clear evidence of ethical approval? Were there any specific ethical considerations around the partnership component? Were there any specific ethical considerations about the music component?
**What was the health focus of the** **study?**	Was the focus on physical/mental health? Was the focus on prevention, treatment, recovery, promotion?
**Was there an evaluation of the use** **of music?**	What methods were used to evaluate the use of music? Was it a process or retrospective evaluation? Was the evaluation positive/negative/mixed?
**Limitations/quality Issues**	Were there any reported limitations or quality issues? (not a critical appraisal)

Edited from JBI Reviewer’s Manual, 11.2.7 Data extraction ( Nittas *et al.*, 2018; Peters *et al.*, 2017). ABR: arts-based research.

### Stage 5: Collating, summarising and reporting the results

As recommended by the JBI Reviewer’s Manual ( Peters *et al.*, 2017), a narrative summary mapping the findings from the extracted data will complement the tabulated results and describe how they relate to the review’s key question and objectives. As suggested by Levac *et al.*, (2010), we will engage in thematic construction, using qualitative descriptive methods to provide an overview of the breadth of the literature. Findings will be organised into thematic categories such as aims, methodological design, key findings, and gaps in the literature. We will consider the meaning of the results in terms of the broader implications for research, policy, and practice in the context of music as an arts-based method in migrant health research.

### Stage 6: Consultation

As suggested by Levac *et al.*, (2010), we will include consultation with stakeholders to add methodological rigour to this study. This scoping review represents the work of the PART-IM (Participatory and Arts-Based Methods Involving Migrants in Health Research) research cluster which is part of the Health Research Institute, Public and Patient Involvement capacity building theme at the University of Limerick. The research cluster brings together arts-based and participatory scholars from medicine, nursing & midwifery, and the performing arts, as well as a leading NGO for migrants. These stakeholders have expertise to inform our scoping review. They have been involved from the beginning in its planning, have commented on this protocol and will be invited to discuss preliminary findings, validate findings and inform ideas about future research.

## Ethics and dissemination

### Ethics

Ethical approval is not required for this scoping review.

### Dissemination

This study integrates collaborative knowledge-sharing through the involvement of key stakeholders in a process of discussion and consultation. In addition, findings will be disseminated through academic conferences and peer review publications focused on participatory arts-based health research in the context of national and international migrant health research and policy.

## Study status

Database searches have been completed. Titles and abstracts have been screened and full texts are being assessed against the eligibility criteria.

## Data availability

No data are associated with this article.
