# Supplementary material for: The use of music as an arts-based method in migrant health research: a scoping review protocol
Source: HRB Open Res. 2020 Oct 12;3:75. [Version 1] doi: 10.12688/hrbopenres.13121.1 (PMC8063534; doi:10.12688/hrbopenres.13121.1)
Supplement: Peer review history

# Peer review: Reviewer response for version 1

**DOI:** 10.21956/hrbopenres.14230.r29264

**Published:** 2021-04-21

**Authors:** Saras Henderson

**Affiliations:** 1 School of Nursing and Midwifery, Griffith University, Southport, Australia

This paper provides a scoping review to ascertain the extent to which music is used as an arts-based research method for heath research in migrant communities. The authors present a robust case for this review highlighting the dearth of available data in this area. The authors show how music has been reported as being an intervention outcome rather than being the research method. The protocol and eligibility criteria for publication are described. How articles were assessed for quality would be useful.

Is the study design appropriate for the research question?

Yes

Is the rationale for, and objectives of, the study clearly described?

Yes

Are sufficient details of the methods provided to allow replication by others?

Yes

Are the datasets clearly presented in a useable and accessible format?

Not applicable

Reviewer Expertise:

Health research in culturally and linguistically diverse communities including refugees. Health service research in preventing life style and chronic diseases in the community.

I confirm that I have read this submission and believe that I have an appropriate level of expertise to confirm that it is of an acceptable scientific standard.

# Peer review: Reviewer response for version 1

**DOI:** 10.21956/hrbopenres.14230.r28099

**Published:** 2020-11-05

**Authors:** Kari Veblen

**Affiliations:** 1 Don Wright Faculty of Music, University of Western Ontario, London, ON, Canada

Thank you for the opportunity to review this paper.

 This article describes the rationale and qualitative method employed in a cross-disciplinary investigation. The study examines music being used as an arts-based research method for health/wellness research in migrant communities. The authors make a convincing case for their inquiry, noting that music is amply documented as a powerful tool for social engagement, connection, and wellness. The use of music as an arts-based method in health research is less clear, although other forms such as theatre and visual arts are established in this use. 

 Using a scoping review based on Levac *et al* (2010), the authors survey current publications against a template of eligibility criteria. While the study collection is completed, the results are still in process of collation, summarization, dissemination.

Is the study design appropriate for the research question?

Yes

Is the rationale for, and objectives of, the study clearly described?

Yes

Are sufficient details of the methods provided to allow replication by others?

Yes

Are the datasets clearly presented in a useable and accessible format?

Not applicable

Reviewer Expertise:

Music education, ethnomusicology, community music, social media and music learning, sociology of music

I confirm that I have read this submission and believe that I have an appropriate level of expertise to confirm that it is of an acceptable scientific standard.
